# Comparative evaluation of trimethoprim-sulfonamide ratios and synergistic interactions against porcine respiratory pathogens

**DOI:** 10.3389/fvets.2026.1805735

**Published:** 2026-04-10

**Authors:** Patrik Mag, Ulrich Klein, Wouter Depondt, Imre Sándor Piross, Réka Tóth, Zoltán Somogyi, Dóra Kovács, Ádám Kerek, Ákos Jerzsele

**Affiliations:** 1 Department of Pharmacology and Toxicology, University of Veterinary Medicine Budapest, Budapest, Hungary; 2 National Laboratory of Infectious Animal Diseases, Antimicrobial Resistance, Veterinary Public Health, and Food Chain Safety, University of Veterinary Medicine Budapest, Budapest, Hungary; 3 Huvepharma NV, Antwerp, Belgium; 4 Department of Biostatistics, University of Veterinary Medicine Budapest, Budapest, Hungary; 5 Lendület Ecosystem Services Research Group, Institute of Ecology and Botany, HUN-REN Centre for Ecological Research, Vácrátót, Hungary; 6 Department of Microbiology and Infectious Diseases, University of Veterinary Medicine Budapest, Budapest, Hungary; 7 Pig-Care Ltd., Bodajk, Hungary

**Keywords:** antimicrobial interactions, FICI, porcine respiratory pathogens, potentiated sulfonamides, trimethoprim-sulfonamide synergy

## Abstract

**Introduction:**

Potentiated sulfonamides are widely used in the treatment of porcine respiratory disease complex, but their synergistic activity depends on pathogen-related and pharmacodynamic factors, including the trimethoprim-to-sulfonamide ratio. This study aimed to systematically evaluate the effect of different ratios on in vitro antibacterial interactions against major porcine respiratory pathogens.

**Methods:**

Trimethoprim was combined with sulfamethoxazole, sulfachloropyridazine, or sulfadiazine at four mass ratios (1:5, 1:10, 1:19, 1:40). Minimum inhibitory concentrations were determined using broth microdilution according to CLSI guidelines. Drug interactions were quantified using the fractional inhibitory concentration index, and a linear mixed-effects model was applied to assess the effects of bacterial species, compound combination, and ratio.

**Results:**

The strongest and most consistent synergistic activity was observed at the 1:5 ratio across most pathogen–drug combinations. Increasing the proportion of the sulfonamide generally reduced synergism and occasionally resulted in antagonistic interactions, particularly in Glaesserella parasuis. Differences between sulfonamides were limited, although sulfachloropyridazine showed higher intrinsic activity against Actinobacillus pleuropneumoniae and G. parasuis, and greater efficacy in combination with trimethoprim against Pasteurella multocida. Statistical analysis confirmed significant effects of both drug ratio and combination on interaction outcomes.

**Discussion:**

These findings demonstrate that synergism between trimethoprim and sulfonamides is strongly ratio-dependent and cannot be reliably characterized using a single fixed ratio. Ratio-specific evaluation may therefore support more rational antimicrobial use in veterinary medicine.

## 1 Introduction

Porcine respiratory disease complex (PRDC) represents a major animal health and economic problem worldwide (1, 2). The condition primarily affects weaned piglets and growing–finishing pigs, and its development is driven by the combined effects of multiple predisposing factors and pathogens. Primary pathogens include porcine reproductive and respiratory syndrome virus (PRRSV), swine influenza virus (SIV), porcine circovirus type 2 (PCV-2), as well as *Mycoplasma hyopneumoniae*, *Mycoplasma hyorhinis* and *A. pleuropneumoniae*. The clinical course is frequently exacerbated by secondary bacterial infections, most notably *P. multocida*, *Streptococcus suis* and *G. parasuis* (3, 4). Owing to its complex etiology, the treatment of PRDC is challenging, and the use of antibiotics is often unavoidable.

A well-established synergistic interaction exists between sulfonamides and diaminopyrimidines. Sulfonamides inhibit the enzyme dihydropteroate synthase, whereas trimethoprim interferes with folic acid synthesis by blocking dihydrofolate reductase. As tetrahydrofolic acid plays a key role in re-initiating the folate synthesis cycle through metabolic feedback mechanisms, inhibition by trimethoprim further enhances the activity of sulfonamides, resulting in mutual synergism (5).

In commercially available potentiated sulfonamide formulations, the trimethoprim-sulfonamide ratio is most commonly 1:5 (6, 7). In human medicine, the frequently cited 1:19 trimethoprim-sulfonamide ratio originates from pharmacokinetic considerations, as the relatively similar elimination half-lives of the two components result in stable plasma concentration proportions approximating this value following administration (8, 9). Consequently, this ratio has historically been regarded as pharmacokinetically favourable in humans.

In contrast, species-specific pharmacokinetic differences substantially influence the *in vivo* concentration ratio in food-producing animals. Following administration in pigs, trimethoprim distributes more extensively into tissues due to its lipophilic properties, whereas sulfonamides are largely confined to extracellular compartments (6). Differences in distribution volume and clearance between the two components result in time-dependent changes in their plasma concentration ratio rather than a stable fixed proportion (9). Population pharmacokinetic modelling indicates that a dose ratio of 1:5 does not consistently yield a constant *in vivo* ratio in pigs, and that free plasma concentration ratios vary considerably over time and between individuals depending on the sulfonamide and duration after administration (10). Therefore, the 1:19 ratio should be regarded as a historically proposed reference value rather than a universally achieved *in vivo* proportion in pigs.

Based on earlier *in vitro* investigations, proportions close to this 1:19 ratio have been reported to produce favourable interaction profiles for specific bacterial species (9, 11). In some publications, 1:20 trimethoprim-sulfonamide ratio has been theoretically proposed as advantageous in the context of sequential inhibition of the folate synthesis pathway (6, 12, 13). However, such recommendations were derived from particular experimental settings and should not be interpreted as indicating a universally optimal ratio across pathogens or clinical conditions, as synergism is influenced by species-specific susceptibility patterns and the relative MIC values of the individual components (9, 14).

In the present study, the selected ratios (1:5, 1:10, 1:19 and 1:40) were chosen to represent a structured pharmacodynamic spectrum rather than to test a presumed optimal proportion. The 1:5 ratio reflects the composition of most commercial formulations, the 1:19 ratio approximates historically reported plasma proportions, the 1:10 ratio serves as an intermediate transitional condition, and the 1:40 ratio represents a sulfonamide-dominant setting to explore the broader range of ratio-dependent interaction patterns.

With regard to the standardization of antimicrobial susceptibility testing, EUCAST has published MIC distribution data exclusively for the 1:19 trimethoprim-sulfamethoxazole combination (15). Similarly, CLSI has established clinical breakpoints for trimethoprim-sulfamethoxazole only for specific human pathogens and primarily at this standard 1:19 ratio (16). Accordingly, laboratory susceptibility testing is typically performed using this ratio (17–19). Species-specific clinical breakpoints for trimethoprim-sulfonamide combinations against porcine respiratory pathogens are not defined.

The number of studies investigating the synergism of potentiated sulfonamides remains limited. Rattanapanadda et al. (20) demonstrated both *in vitro* and *in vivo* synergism between florfenicol and thiamphenicol against *A. pleuropneumoniae* and *P. multocida*, and further suggested that florfenicol may exhibit synergistic interactions with tetracyclines and erythromycin. This is particularly noteworthy, as synergism between antibiotics sharing the same mechanism of action is rarely observed (21, 22).

The aim of the present study was to investigate the interactions between the components of three different potentiated sulfonamides—trimethoprim-sulfamethoxazole, trimethoprim-sulfachloropyridazine and trimethoprim-sulfadiazine—against *P. multocida*, *S. suis*, *A. pleuropneumoniae* and *G. parasuis* isolates obtained from pigs with respiratory disease, using combinations at different ratios (1:5, 1:10, 1:19 and 1:40). To our knowledge, this is the first study to specifically analyse the synergistic effects of these combinations against pathogens associated with PRDC.

## 2 Materials and methods

### 2.1 Antimicrobial agents, bacteria

Trimethoprim (TMP), sulfamethoxazole (SMX), sulfachloropyridazine (SCP) and sulfadiazine (SD) used in the experiments were purchased from Sigma-Aldrich (St. Louis, MO, United States). All active substances were analytical reference standards with a purity of >98%.

The *P. multocida* (*n* = 12), *S. suis* (*n* = 14), *A. pleuropneumoniae* (*n* = 13) and *G. parasuis* (*n* = 10) strains included in the study were isolates from diseased animals collected between 2018 and 2022 within the framework of the VetPath European monitoring program in Denmark, Germany, Netherlands, Spain, United Kingdom. The isolates originated from harmonized diagnostic sampling of diseased or recently deceased animals presenting acute clinical signs consistent with respiratory or systemic infection. Samples were obtained primarily from lung tissue and, in some cases, from brain tissue collected during routine diagnostic investigation of systemic disease (e.g., suspected meningitis). To ensure epidemiological independence and to avoid overrepresentation of individual outbreaks, only one isolate per outbreak per farm was retained for further characterization. Animals had not been exposed to antimicrobial treatment for at least 15 days prior to sampling. In all cases, species identification was performed using MALDI-TOF mass spectrometry (MALDI-TOF MS) at the Department of Microbiology and Infectious Diseases, University of Veterinary Medicine Budapest.

### 2.2 *In vitro* susceptibility testing

Minimum inhibitory concentration (MIC) values of the individual active substances against each bacterial species were determined using the broth microdilution method, in accordance with the relevant Guidelines of the Clinical and Laboratory Standards Institute (23). MICs were determined for the individual compounds (TMP, SMX, SCP and SD), as well as for the potentiated sulfonamide combinations (TMP-SMX, TMP-SCP and TMP-SD). The trimethoprim–sulfonamide ratios (1:5, 1:10, 1:19 and 1:40) were predefined to represent a structured concentration spectrum encompassing the formulation ratio (1:5), the historically adopted reference ratio (1:19), an intermediate condition (1:10), and a sulfonamide-dominant setting (1:40).

Brief method description: two-fold serial dilutions of the tested active substances and their combinations were prepared in cation-adjusted Mueller–Hinton II broth (CAMHB) (Biolab Ltd., Budapest, Hungary) in 96-well microplates (96-well BRANDplates—F—pureGrade S, VWR International, Radnor, Pennsylvania, United States). The broth was supplemented with 10 μg/mL nicotinamide adenine dinucleotide (*β* NAD, Sigma-Aldrich, St. Louis, MO, United States) to meet the V-factor requirements of *A. pleuropneumoniae* and *G. parasuis* strains (24, 25). To ensure methodological consistency across all tested species and combinations, NAD supplementation was applied uniformly to all isolates.

The bacterial inoculum was prepared in the same NAD-supplemented CAMHB. Bacterial suspensions were inoculated into the wells to achieve a final concentration of 5 × 10^5^ colony-forming units (CFU/mL). Plates were incubated at 37 °C for 16–18 h under 5% CO_2_-enriched conditions. These incubation conditions were applied uniformly to all species to ensure adequate growth of fastidious pathogens while maintaining comparability across tested ratios. Following incubation, the MIC was defined as the lowest concentration at which no visible bacterial growth was observed. All MIC determinations were performed in triplicate.

CLSI species-specific clinical breakpoints are not defined for trimethoprim-sulfonamide combinations against the investigated porcine respiratory pathogens; therefore, categorical S/I/R interpretation was not performed. Baseline susceptibility was characterized using MIC_50_, MIC_90_ and MIC ranges.

### 2.3 Determination of FICI value

Following the determination of MIC values for the individual active substances and the different combination ratios, the extent of synergistic interactions between the compounds was assessed using the fractional inhibitory concentration index (FICI). The FICI value was calculated according to the following equation:


$$\text{FICI}=\frac{C_{A}}{{\mathrm{MIC}}_{A}}+\frac{C_{B}}{{\mathrm{MIC}}_{B}}$$

where MIC_A_ and MIC_B_ are the minimum inhibitory concentrations of antibacterial agents A and B, and C_A_ and C_B_ are the concentrations that together inhibit bacterial growth. The following five interactions between two ingredients in a combination can be assumed: synergistic (FICI ≤ 0.5), partially synergistic (0.5 < FICI < 1), additive (FICI = 1), indifferent (1 < FICI < 4) or antagonistic (FICI ≥ 4) (26, 27).

For each bacterial species and combination ratio, descriptive statistical parameters of the FICI values were calculated. In addition to the arithmetic mean, the median and interquartile range (IQR) were determined to provide a robust characterization of central tendency and dispersion. The IQR was defined as the range between the first and third quartiles (Q1–Q3), corresponding to the 25th and 75th percentiles of the ordered FICI values. Results are presented as mean and median (IQR).

### 2.4 Exploratory analysis of the relationship between combination MIC and FICI values

In an additional exploratory analysis, isolate-level MIC values of the potentiated sulfonamide combinations were evaluated together with the corresponding FICI values in a paired manner for each bacterial strain, antimicrobial combination, and tested ratio. This analysis was based on the MIC values of the trimethoprim-sulfonamide combinations presented in Supplementary Tables S2–S4 and the corresponding isolate-level FICI values presented in Supplementary Tables S5–S7.

The aim of this exploratory approach was to assess whether lower or higher MIC values of the tested combinations were associated with systematically different interaction patterns, as reflected by the magnitude of the FICI values and their categorical interpretation. Because no species-specific clinical breakpoints are available for these combinations against the investigated porcine respiratory pathogens, this analysis was descriptive in nature and no categorical susceptibility interpretation was applied to MIC values. FICI values were interpreted according to the same criteria used throughout the study: synergistic (FICI ≤ 0.5), partially synergistic (0.5 < FICI < 1), additive (FICI = 1), indifferent (1 < FICI < 4), and antagonistic (FICI ≥ 4).

### 2.5 Statistical comparison of FICI values

The log-transformed FICI values were analysed using a linear mixed-effects model (28). The model included bacterial species, antimicrobial combination, and compound ratio as fixed effects, with all two- and three-way interactions. To account for repeated measurements across different compound ratios, a random intercept for bacterial strain was included.

Model assumptions were assessed visually using Q–Q plots of the random effects and residuals, fitted-vs-residual plots, and scale-location plots. Model fit was evaluated by comparing observed log-transformed FICI values to simulated values from the fitted model, and by comparing fitted values (back-transformed to the response scale) to observed means by bacterial species, antimicrobial agent, and ratio.

Although descriptive statistics are additionally reported as mean and median (interquartile range, IQR) to provide a robust characterization of central tendency and dispersion, inferential analyses were conducted within the linear mixed-effects modelling framework. Under the assumptions of the model, the expected value (mean) of the log-transformed response is estimated directly. Model diagnostics indicated approximate normality and homoscedasticity of residuals on the log scale, supporting the appropriateness of mean-based inference despite right-skewness on the original measurement scale.

For hypothesis testing and effect interpretation, Wald *χ*^2^ tests were used to assess the significance of main terms and interactions. Estimated marginal means (EMMs) were then calculated for each compound ratio within each bacteria-antimicrobial agent combination. EMMs were calculated on the response scale by back-transforming from the log-transformed values predicted by the model, allowing direct comparison of mean FICI values across all treatment combinations while accounting for strain-level variation through the random intercept. To test for significant differences in FICI values between compound ratios, five custom pairwise contrasts were applied: 1:10 versus 1:5, 1:19 versus 1:5, 1:40 versus 1:5, 1:19 versus 1:10, and 1:40 versus 1:19. These contrasts were computed separately for each bacterial species and antimicrobial combination, with Holm adjustment applied to correct for multiple comparisons.

All analyses were performed in R 4.4.3 using the following packages: tidyverse 2.0.0, readxl 1.4.5, glmmTMB 1.1.11, car 3.1-3, DHARMa 0.4.7, emmeans 1.11.1, knitr 1.50, kableExtra 1.4.0., and writexl 1.5.4.

## 3 Results

### 3.1 Determination of individual MIC values for trimethoprim and sulfonamides

Trimethoprim exhibited the highest activity against *A. pleuropneumoniae* strains (MIC_50_ = 8 μg/mL, MIC_90_ = 16 μg/mL), where MIC_50_ and MIC_90_ values differed by only one dilution step and the MIC range remained relatively narrow compared with other species. Moderate susceptibility was observed among *G. parasuis* isolates, whereas *P. multocida* and *S. suis* strains showed only marginal susceptibility, with MIC_50_ and MIC_90_ values often identical but accompanied by broad MIC ranges, indicating heterogeneous baseline susceptibility. A similar overall trend was observed for the sulfonamide compounds.

Among the sulfonamides, *A. pleuropneumoniae* strains were most susceptible to SCP (MIC_50_ = 40 μg/mL, MIC_90_ = 80 μg/mL). In contrast, several species-compound combinations demonstrated substantial dispersion, reflected by multiple dilution-step differences between MIC_50_ and MIC_90_ values and wide MIC ranges extending to the highest tested concentrations. Overall, isolates were slightly more susceptible to SMX than to SD. Detailed MIC_50_, MIC_90_ and range values are summarised in Table 1. Individual MIC values for all bacterial isolates are presented in Supplementary Table S1.

**Table 1:** MIC_50_ and MIC_90_ values and MIC ranges (μg/mL) of the sole agents trimethoprim (TMP), sulfamethoxazole (SMX), sulfachloropyridazine (SCP) and sulfadiazine (SD) against *Pasteurella multocida* (*P. multocida*), *Streptococcus suis* (*S. suis*), *Actinobacillus pleuropneumoniae* (*A. pleuropneumoniae*) and *Glaesserella parasuis* (*G. parasuis*) strains.

Bacterium	MIC values	TMP	SMX	SCP	SD
*P. multocida* (*n* = 12)	MIC_50_ MIC_90_ Range	256 256 128–256	5,120 5,120 5,120	640 1,280 80–1,280	5,120 >5,120 2,560–>5,120
*S. suis* (*n* = 14)	MIC_50_ MIC_90_ Range	256 256 64–256	640 640 160–2,560	160 1,280 <10–2,560	1,280 >5,120 160–>5,120
*A. pleuropneumoniae* (*n* = 13)	MIC_50_ MIC_90_ Range	8 16 0.5–128	80 5,120 40–5,120	40 80 20–160	2,560 5,120 80–5,120
*G. parasuis* (*n* = 10)	MIC_50_ MIC_90_ Range	64 128 2–128	1,280 5,120 10–5,120	80 320 10–320	1,280 2,560 10–5,120

### 3.2 MIC determination of potentiated sulfonamides at different ratios

For the different bacterial species, increasing the proportion of sulfonamide in the combinations generally resulted in stable or decreasing MIC values, although the magnitude of change varied among species and combinations. A consistent reduction across increasing sulfonamide ratios was most evident for *A. pleuropneumoniae* and, to a lesser extent, for *S. suis*, whereas in *P. multocida* MIC values remained largely unchanged across ratios for TMP-SMX and TMP-SD. The only clear exception was observed for the TMP-SD combination against *G. parasuis*, where no consistent decreasing trend was apparent.

Potentiated sulfonamides, similarly to the individual compounds, showed the highest activity against *A. pleuropneumoniae* strains, with MIC_90_ values ranging from 2 to 4 μg/mL across all three combinations and ratio conditions, accompanied by relatively narrow MIC ranges compared with the other species. Moderate activity was observed against *G. parasuis* isolates, whereas *P. multocida* and *S. suis* strains exhibited substantially higher MIC_90_ values and broad MIC ranges, indicating heterogeneous baseline susceptibility.

Among the different combinations, a notable difference in potency was observed primarily for *P. multocida*, where the TMP-SCP combination exhibited lower MIC_50_ and MIC_90_ values compared with TMP-SMX and TMP-SD across several ratio conditions. MIC values for the different ratio combinations, including MIC_50_, MIC_90_ and full MIC ranges, are summarised in Table 2. Individual MIC values for all bacterial isolates are presented in Supplementary Tables S2–S4.

**Table 2:** MIC_50_ and MIC_90_ values and MIC ranges (μg/mL) of trimethoprim-sulfamethoxazole (TMP-SMX), trimethoprim-sulfachloropyridazine (TMP-SCP) and trimethoprim-sulfadiazine (TMP-SD) combinations in ratios of 1:5, 1:10, 1:19, and 1:40 against *Pasteurella multocida* (*P. multocida*), *Streptococcus suis* (*S. suis*), *Actinobacillus pleuropneumoniae* (*A. pleuropneumoniae*) and *Glaesserella parasuis* (*G. parasuis*) strains.

Bacterium	Antimicrobial agent	MIC values	1:5	1:10	1:19	1:40
*P. multocida* (*n* = 12)	TMP-SMX	MIC_50_ MIC_90_ Range	256 256 128–256	256 256 256	256 256 64–256	128 256 128–256
	TMP-SCP	MIC_50_ MIC_90_ Range	64 64 32–128	32 64 32–128	16 32 16–128	16 32 4–128
	TMP-SD	MIC_50_ MIC_90_ Range	256 256 256	256 256 256	256 256 256	256 256 128–256
*S. suis* (*n* = 14)	TMP-SMX	MIC_50_ MIC_90_ Range	32 128 2–256	32 64 2–256	16 64 2–256	8 64 2–256
	TMP-SCP	MIC_50_ MIC_90_ Range	32 256 4–256	16 64 2–256	16 64 2–256	8 64 2–128
	TMP-SD	MIC_50_ MIC_90_ Range	16 256 4–256	16 128 4–256	16 64 2–256	16 64 2–256
*A. pleuropneumoniae* (*n* = 13)	TMP-SMX	MIC_50_ MIC_90_ Range	2 4 0.5–4	2 4 0.5–4	1 2 0.5–4	1 2 0.25–2
	TMP-SCP	MIC_50_ MIC_90_ Range	2 4 0.5–8	2 4 0.5–8	2 2 0.25–8	0.5 2 0.25–2
	TMP-SD	MIC_50_ MIC_90_ Range	2 4 0.5–8	2 4 1–4	2 4 0.5–8	1 2 0.5–4
*G. parasuis* (*n* = 10)	TMP-SMX	MIC_50_ MIC_90_ Range	2 32 1–64	2 32 1–32	2 16 1–32	2 16 0.5–32
	TMP-SCP	MIC_50_ MIC_90_ Range	2 32 1–32	4 16 1–64	2 16 1–32	2 8 0.5–8
	TMP-SD	MIC_50_ MIC_90_ Range	4 32 2–64	4 64 2–64	2 32 1–32	2 32 1–32

#### 3.2.1 Determination of FICI value

For most tested bacterial species, increasing the proportion of the sulfonamide component was generally associated with higher mean FICI values, although the pattern was not strictly monotonic across all combinations and ratios. This tendency was most evident for *G. parasuis*, where mean FICI values increased progressively with higher sulfonamide proportions in all three combinations. In contrast, deviations from this pattern were observed for *P. multocida* and *S. suis*, particularly in the TMP-SCP combinations at 1:19 and 1:40 ratios, where mean values did not consistently increase. For *A. pleuropneumoniae*, mean FICI values for TMP-SCP and TMP-SD combinations showed moderate variation across ratios, with a slight decrease at higher sulfonamide proportions in selected cases.

When considering both mean and median (IQR) values, the lowest central FICI estimates were generally observed at the 1:5 ratio across species and combinations. The only clear exception was the TMP-SCP combination against *A. pleuropneumoniae*, where the lowest mean FICI value occurred at the 1:40 ratio; in contrast, the median (IQR) indicated the lowest FICI at the 1:5 ratio. Notably, synergistic interactions (FICI < 1) were detected more frequently when assessed on the basis of median values than when evaluated using mean FICI estimates. Among the individual species, *G. parasuis* strains exhibited the most pronounced synergistic interactions, reflected by consistently low mean and median FICI values. Mean and median (IQR) FICI values for all combinations and ratios are summarised in Table 3. Individual FICI values for all bacterial isolates are presented in Supplementary Tables S5–S7.

**Table 3:** Average and median (interquartile range, IQR) FICI values for combinations of trimethoprim–sulfamethoxazole (TMP–SMX), trimethoprim–sulfachloropyridazine (TMP–SCP) and trimethoprim–sulfadiazine (TMP–SD) at ratios of 1:5, 1:10, 1:19 and 1:40 against *Pasteurella multocida* (*P. multocida*), *Streptococcus suis* (*S. suis*), *Actinobacillus pleuropneumoniae* (*A. pleuropneumoniae*) and *Glaesserella parasuis* (*G. parasuis*) strains.

Bacterium	Antimicrobial agent	FICI	1:5	1:10	1:19	1:40
*P. multocida* (*n* = 12)	TMP-SMX	Mean	1.19	1.58	1.61	1.92
		Median (IQR)	1.25 1.25–1.25	1.50 1.50–1.50	1.95 0.98–1.95	1.50 1.50–3.00
	TMP-SCP	Mean	1.50	2.53	2.20	4.06
		Median (IQR)	*0.88* 0.38–2.25	1.00 0.63–4.25	1.08 0.60–3.86	1.13 1.06–8.06
	TMP-SD	Mean	1.31	1.54	1.95	2.75
		Median (IQR)	1.25 1.13–1.25	1.50 1.25–1.50	1.95 1.48–1.95	3.00 2.00–3.00
*S. suis* (*n* = 14)	TMP-SMX	Mean	1.03	1.45	1.92	3.30
		Median (IQR)	*0.38* 0.19–1.69	*0.63* 0.31–3.19	*0.54* 0.38–3.44	1.06 0.80–2.08
	TMP-SCP	Mean	4.35	6.50	7.92	7.77
		Median (IQR)	*0.88* 0.31–3.06	*0.81* 0.27–5.56	1.99 0.50–9.06	2.05 1.01–6.08
	TMP-SD	Mean	**0.81**	**0.91**	**0.96**	1.82
		Median (IQR)	*0.19* 0.09–0.66	*0.31* 0.13–1.58	*0.40* 0.09–1.71	*0.84* 0.15–3.08
*A. pleuropneumoniae* (*n* = 13)	TMP-SMX	Mean	1.11	1.13	1.15	1.19
		Median (IQR)	*0.38* 0.19–2.00	*0.53* 0.22–1.51	*0.49* 0.24–1.61	1.00 0.31–1.75
	TMP-SCP	Mean	1.23	1.38	1.32	1.03
		Median (IQR)	*0.50* 0.44–1.77	*0.75* 0.56–1.38	1.20 0.87–1.58	1.06 0.56–1.69
	TMP-SD	Mean	1.04	1.34	1.07	1.05
		Median (IQR)	*0.31* 0.27–1.51	*0.38* 0.28–2.01	*0.49* 0.18–1.88	*0.50* 0.17–2.01
*G. parasuis* (*n* = 10)	TMP-SMX	Mean	**0.44**	**0.57**	**0.74**	1.33
		Median (IQR)	*0.33* 0.05–1.00	*0.36* 0.05–1.02	*0.39* 0.06–0.96	*0.42* 0.05–1.13
	TMP-SCP	Mean	**0.67**	1.28	1.42	1.61
		Median (IQR)	*0.52* 0.14–1.06	*0.91* 0.47–2.13	1.22 0.48–1.96	0.81 0.52–1.50
	TMP-SD	Mean	**0.53**	**0.85**	1.12	2.07
		Median (IQR)	*0.31* 0.08–0.75	*0.38* 0.12–1.01	*0.38* 0.08–0.74	*0.45* 0.09–1.03

Bold indicates values suggesting positive interaction based on mean FICI (FICI <1). Italics indicates values suggesting positive interaction based on median FICI (FICI <1).

A more detailed picture of the synergistic interactions between the active substances for each bacterial species can be obtained by examining the distribution of isolates across the different FICI categories rather than relying solely on mean or median values (see Table 4).

**Table 4:** Distribution of synergistic (S), partial synergistic (PS), additive (Ad), indifferent (I) and antagonistic (An) interactions in the *Pasteurella multocida* (*P. multocida*), *Streptococcus suis* (*S. suis*), *Actinobacillus pleuropneumoniae* (*A. pleuropneumoniae*) and *Glaesserella parasuis* (*G. parasuis*) strains for combinations of trimethoprim-sulfamethoxazole (TMP-SMX), trimethoprim-sulfachloropyridazine (TMP-SCP) and trimethoprim-sulfadiazine (TMP-SD) at ratios of 1:5, 1:10, 1:19 and 1:40, by percentage.

Bacteria organism	Antimicrobial agent	Ratio	S	PS	Ad	I	An
*P. multocida* (*n* = 12)	TMP-SMX	1:5	0.0	8.3	0.0	91.7	0.0
		1:10	0.0	0.0	0.0	100.0	0.0
		**1:19**	0.0	33.3	0.0	66.7	0.0
		1:40	0.0	0.0	0.0	100.0	0.0
	TMP-SCP	**1:5**	41.7	8.3	8.3	25.0	16.7
		1:10	8.3	41.7	0.0	8.3	41.7
		1:19	8.3	33.3	0.0	50.0	8.3
		1:40	16.7	0.0	0.0	50.0	33.3
	TMP-SD	**1:5**	0.0	0.0	0.0	100.0	0.0
		1:10	0.0	0.0	0.0	100.0	0.0
		1:19	0.0	0.0	0.0	100.0	0.0
		1:40	0.0	0.0	0.0	91.7	8.3
*S. suis* (*n* = 14)	TMP-SMX	**1:5**	57.1	7.1	0.0	28.6	7.1
		1:10	35.7	28.6	0.0	14.3	21.4
		1:19	35.7	28.6	0.0	21.4	14.3
		1:40	14.3	14.3	0.0	57.1	14.3
	TMP-SCP	**1:5**	35.7	14.3	0.0	28.6	21.4
		1:10	35.7	14.3	0.0	28.6	21.4
		1:19	28.6	7.1	0.0	35.7	28.6
		1:40	14.3	7.1	0.0	35.7	42.9
	TMP-SD	**1:5**	71.4	0.0	0.0	21.4	7.1
		1:10	71.4	0.0	0.0	21.4	7.1
		1:19	50.0	21.4	0.0	28.6	0.0
		1:40	35.7	14.3	0.0	28.6	21.4
*A. pleuropneumoniae* (*n* = 13)	TMP-SMX	**1:5**	69.2	0.0	0.0	15.4	15.4
		1:10	46.2	15.4	7.7	15.4	15.4
		1:19	53.8	7.7	0.0	23.1	15.4
		1:40	38.5	7.7	0.0	38.5	15.4
	TMP-SCP	**1:5**	61.5	7.7	0.0	15.4	15.4
		1:10	23.1	30.8	0.0	30.8	15.4
		1:19	7.7	15.4	0.0	76.9	0.0
		1:40	15.4	30.8	0.0	53.8	0.0
	TMP-SD	1:5	61.5	7.7	0.0	15.4	15.4
		**1:10**	69.2	0.0	0.0	15.4	15.4
		1:19	61.5	7.7	0.0	15.4	15.4
		1:40	53.8	7.7	0.0	30.8	7.7
*G. parasuis* (*n* = 10)	TMP-SMX	**1:5**	50.0	20.0	10.0	20.0	0.0
		1:10	50.0	20.0	0.0	30.0	0.0
		1:19	50.0	30.0	0.0	20.0	0.0
		1:40	50.0	10.0	0.0	30.0	10.0
	TMP-SCP	**1:5**	50.0	10.0	10.0	30.0	0.0
		1:10	30.0	20.0	0.0	40.0	10.0
		1:19	30.0	0.0	0.0	70.0	0.0
		1:40	10.0	40.0	0.0	40.0	10.0
	TMP-SD	**1:5**	60.0	20.0	0.0	20.0	0.0
		1:10	50.0	10.0	10.0	20.0	10.0
		1:19	50.0	30.0	0.0	10.0	10.0
		1:40	50.0	10.0	10.0	20.0	10.0

We marked with bold the lowest ratio at which we detected synergistic interaction in the highest proportion.

In most cases, the strongest synergistic interactions were observed for the 1:5 ratio of the potentiated sulfonamide combinations. Exceptions included the TMP-SMX combination, where the highest proportion of synergistic isolates was observed at the 1:19 ratio for *P. multocida* strains, and the TMP-SD combination, which was most synergistic at the 1:10 ratio for *A. pleuropneumoniae* strains.

For *P. multocida* isolates, an indifferent interaction predominated across most strains, regardless of the compound or ratio. In contrast, synergistic interactions were dominant for the other bacterial species at the tested ratios. Specifically, the TMP-SCP combination showed the highest proportion of synergistic interactions for *P. multocida*, TMP-SMX for *A. pleuropneumoniae*, and TMP-SD for *S. suis* and *G. parasuis* isolates at the 1:5 ratio.

It is important to note that antagonistic interactions were also observed for all bacterial species. These were least pronounced for *P. multocida* and most prominent for *S. suis* strains.

### 3.3 Exploratory relationship between combination MIC values and FICI values

Examination of paired isolate-level MIC and FICI values did not reveal a consistent association between the MIC values of the potentiated sulfonamide combinations and the extent of synergistic interaction. Across the investigated bacterial species, antimicrobial combinations, and tested ratios, isolates with similar combination MIC values frequently exhibited markedly different FICI values, including synergistic, partially synergistic, indifferent, and, in some cases, antagonistic interaction patterns.

In several bacteria-combination settings, lower MIC values coincided with lower FICI values, suggesting more favourable interaction profiles; however, this tendency was not uniform. Conversely, higher MIC values were not consistently associated with either stronger or weaker interaction effects, and in some cases were accompanied by substantial dispersion in FICI values. Overall, the isolate-level data indicate that the magnitude and direction of interaction could not be reliably inferred from combination MIC values alone. The detailed paired MIC and FICI data underlying this exploratory assessment are presented in Supplementary Tables S2–S7.

### 3.4 Statistical modelling of FICI values

The results of the Wald *χ*^2^ tests from the fitted linear mixed-effects model evaluating FICI values indicated that antibacterial combination had a strong and highly significant main effect on FICI values (*p* < 0.0001), as did compound ratio (*p* < 0.0001), indicating that both the identity of the combination and the dosage ratio influenced the degree of synergism. The interaction between bacterial species and antibacterial combination was also significant (*p* < 0.0001), reflecting species-specific responses to different treatments. The main effect of bacterial species was significant as well (*p* = 0.0168), implying detectable differences in baseline susceptibility across species. In contrast, the three-way interaction between bacterial species, antibacterial combination, and dosage ratio was not significant (*p* = 0.8312), meaning no evidence was found that dosage effects differed across bacteria-antibacterial combination pairings.

**Figure 1:**
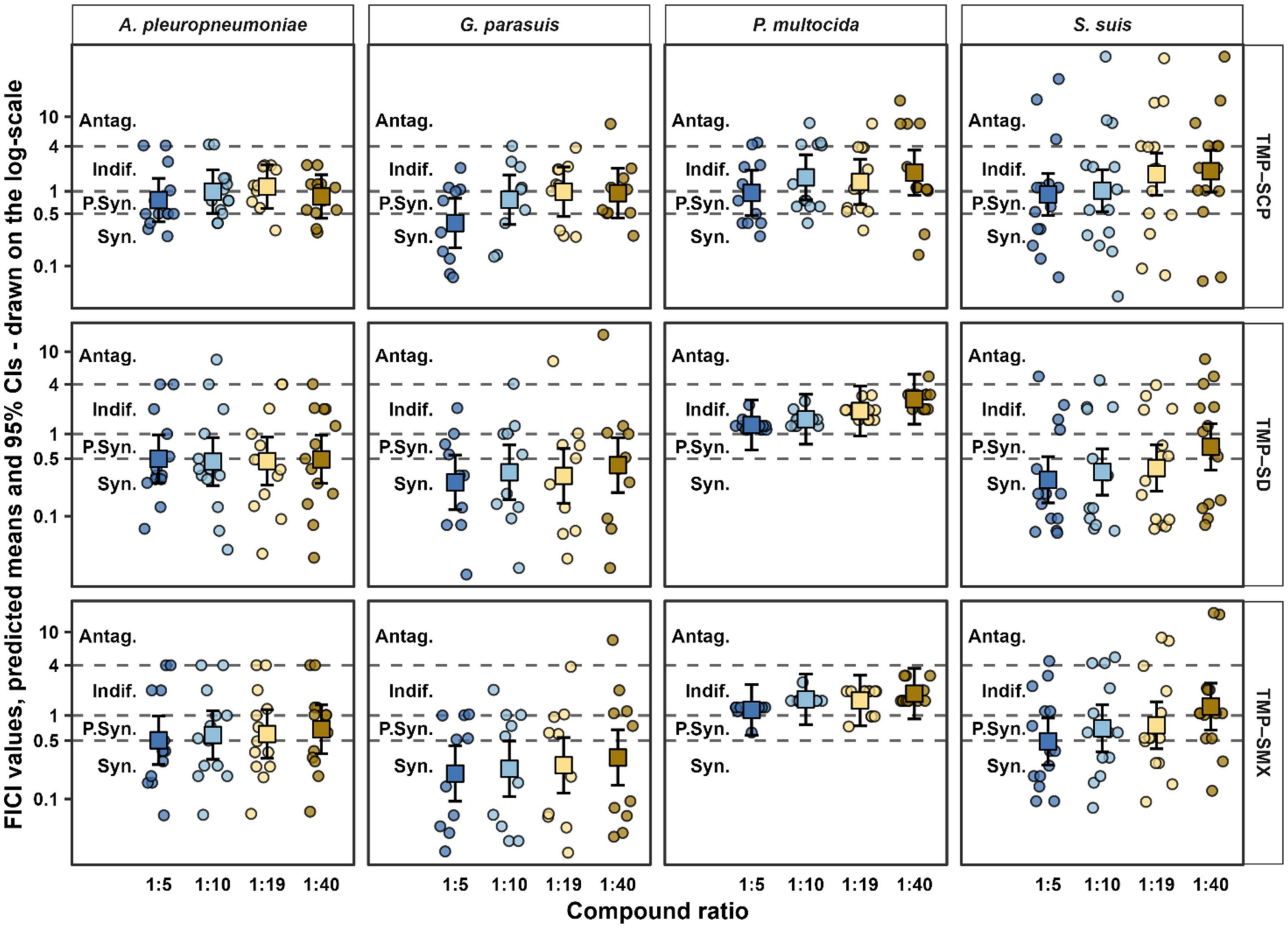
FICI values across compound ratios for three trimethoprim-based (TMP) antibacterial combinations (TMP-sulfachloropyridazine, TMP-sulfadiazine, TMP-sulfamethoxazole) tested against four porcine pathogenic bacteria (*A. pleuropneumoniae*, *G. parasuis*, *P. multocida*, *S. suis*). Small points represent individual observations. Large circles with error bars show the estimated marginal means with 95% confidence intervals (CI) from the fitted linear mixed-effects model. Dashed horizontal lines indicate interpretation thresholds: synergistic (Syn.), partially synergistic (P. Syn.), indifferent (Indif.), and antagonistic (Antag.). Significant pairwise differences between compound ratios are summarised in Table 5, including higher FICI values at 1:40 compared with 1:5 for *S. suis* under all three TMP combinations, and significant increases for *G. parasuis* under TMP-SCP.

The estimated marginal means (EMMs) with 95% confidence intervals (CI) are visualized in Figure 1. The significant (*p* < 0.05) estimated pairwise contrasts between compound ratios for each combination of bacterial species and antibacterial are presented in Table 5. Several significant differences in FICI values were detected between compound ratios, indicating dose-dependent variation in interaction strength for certain bacteria-antibacterial combinations. For *G. parasuis* treated with TMP-SCP, both the 1:19 vs. 1:5 (*p* = 0.0114) and 1:40 vs. 1:5 (*p* = 0.0145) contrasts were significant. A similar dose effect was observed in *S. suis* under TMP–SCP, where FICI values at 1:40 were significantly higher than at 1:5 (*p* = 0.0364). Although observed and modelled results for TMP-SCP against *S. suis* showed similar trends, the model consistently underestimated the mean FICI values in this group.

**Table 5:** Significant estimated pairwise contrasts (*p* < 0.05) between compound ratios (trimethoprim-sulfamethoxazole as TMP-SMX, trimethoprim-sulfachloropyridazine as TMP-SCP and trimethoprim-sulfadiazine as TMP-SD) for each combination of bacterial species and antibacterial agent (calculated from the linear mixed-effects model).

Antibacterial comb.	Bacteria spp.	Compared dosages	Est. ratio between dosages	95% CI LB	95% CI UB	*p*-value
TMP-SCP	*G. parasuis*	1:19/1:5	2.63	1.16	5.95	*p* = 0.0114*
TMP-SCP	*G. parasuis*	1:40/1:5	2.51	1.11	5.69	*p* = 0.0145*
TMP-SCP	*S. suis*	1:40/1:5	2.05	1.03	4.09	*p* = 0.0364*
TMP-SD	*S. suis*	1:40/1:5	2.52	1.26	5.02	*p* = 0.0029**
TMP-SMX	*S. suis*	1:40/1:5	2.62	1.31	5.22	*p* = 0.0017**

The table shows estimated ratios of FICI values between dose levels, with 95% confidence intervals (CI with lower bound, LD and upper bound, UB) and Holm-adjusted *p*-values. Contrasts were calculated within each bacteria-antibacterial agent group using custom linear combinations: 1:10 vs. 1:5, 1:19 vs. 1:5, 1:40 vs. 1:5, 1:19 vs. 1:10, and 1:40 vs. 1:19.

Under TMP-SD treatment, only *S. suis* showed a significant difference between 1:40 and 1:5 (*p* = 0.0029), while *P. multocida* showed marginal support for a similar trend (1:40 vs. 1:5, *p* = 0.0638). For TMP-SMX, a significant contrast was observed for *S. suis* at 1:40 vs. 1:5 (*p* = 0.0017), but no other comparisons reached significance. No significant dosage-related contrasts were detected for *A. pleuropneumoniae* under any of the antibacterial combinations.

## 4 Discussion

The aim of the present study was to comparatively evaluate the synergistic interactions of three different trimethoprim-sulfonamide combinations at varying drug ratios against clinically relevant bacterial pathogens isolated from pigs with respiratory disease. Our findings are consistent with previous observations indicating that the antimicrobial efficacy of potentiated sulfonamides cannot be regarded as a uniform or fixed property; rather, it is pathogen-specific and pharmacodynamically complex, determined by the combined influence of multiple factors (29). Taken together, the present findings indicate that both intrinsic susceptibility patterns and concentration ratios jointly determine the magnitude and consistency of synergistic interactions.

Differences in efficacy among the various sulfonamide components were generally not pronounced. Meaningful differences were observed only for the individual sulfonamide compounds, where SCP proved to be considerably more effective against *A. pleuropneumoniae* and *G. parasuis* strains than SMX and SD. Importantly, this observation was supported not only by MIC_90_ values but also by lower MIC_50_ values and comparatively narrower MIC ranges, suggesting a more homogeneous susceptibility distribution for these species–compound combinations. For the potentiated sulfonamides, a clearly enhanced efficacy of the TMP–SCP combination was observed only for *P. multocida* strains when compared with the other two combinations. The susceptibility patterns observed in this study are consistent with those reported in the literature, indicating that *A. pleuropneumoniae* and *G. parasuis* strains are generally more susceptible to potentiated sulfonamides, whereas increased resistance is more commonly observed among *P. multocida* and *S. suis* isolates (17, 18, 30–33).

One of the most important findings of our study is that, for the majority of bacteria-drug combinations, the strongest and most consistent synergistic interactions were observed at the 1:5 trimethoprim-sulfonamide ratio. This conclusion is supported not only by mean FICI values but, in most settings, also by lower median FICI estimates and comparatively narrow interquartile ranges, indicating that the central tendency of interaction patterns favoured the formulation ratio even when distributional variability was taken into account. This is particularly noteworthy given that, in clinical practice, the 1:19 ratio is generally considered standard, primarily based on pharmacokinetic considerations. Our results align with previous observations indicating that the degree of trimethoprim-sulfonamide synergism is not determined by a fixed concentration ratio, but rather by the relative MIC values of the two components, which can vary considerably between different pathogens (14, 34).

To better contextualise these findings, it is important to consider the biological basis of trimethoprim-sulfonamide interaction. The mechanistic basis of trimethoprim-sulfonamide synergy is the sequential inhibition of the bacterial folate synthesis pathway. Sulfonamides inhibit dihydropteroate synthase, whereas trimethoprim inhibits dihydrofolate reductase, thereby blocking consecutive enzymatic steps required for tetrahydrofolate production (6, 35). Optimal potentiation depends on balanced inhibition of both targets; disproportionate inhibition of one step may attenuate the overall synergistic effect. Consequently, the concentration ratio required for maximal interaction may depend on the intrinsic susceptibility profile and the relative MIC ratio of the two components in a given bacterial species (36).

Furthermore, resistance mechanisms affecting the folate pathway—such as mutations in dihydropteroate synthase gene (*dhps*) or dihydrofolate reductase gene (*dhfr*), altered enzyme affinity, or acquisition of resistance determinants (36, 37)—may modify the contribution of each inhibitory step and thereby influence ratio-dependent interaction patterns. Such biological variability may partly explain the species-specific differences in optimal ratios observed in the present study.

Analysis of FICI values across the different ratios highlighted the dynamic nature of antimicrobial interactions and their sensitivity to the relative concentrations of the components. In several cases, increasing the proportion of the sulfonamide led to higher FICI values, indicating a gradual weakening of the synergistic effect. This phenomenon was particularly pronounced for *G. parasuis* isolates. Odds (38) and Meletiadis et al. (39) emphasize that the interpretation of FICI values is meaningful only when the applied drug ratio is taken into account, and that relying on a single fixed ratio may lead to misleading conclusions.

The presence of occasional high FICI values within otherwise synergistic interaction patterns warrants careful interpretation. Extreme FICI values may arise from isolates exhibiting markedly elevated MIC values for one of the individual components, thereby substantially influencing the fractional contribution of that agent in the FICI calculation. In such situations, the calculated FICI may reflect disproportionate inhibition of a single enzymatic step rather than true biological antagonism (38, 39). Moreover, because FICI values are directly derived from independently determined MIC values of the single agents and their combinations, they are inherently sensitive to minor shifts in MIC determination, particularly when one component exhibits borderline susceptibility. Therefore, isolated extreme values should be interpreted in the context of the overall distribution rather than as evidence of consistent antagonistic interaction.

In several bacteria-combination settings, noticeable differences were observed between mean and median FICI values, accompanied by relatively wide interquartile ranges. This distributional pattern suggests heterogeneity among isolates rather than uniform interaction behaviour. From a biological perspective, such variability may reflect strain-level differences in intrinsic MIC ratios of trimethoprim and the respective sulfonamide, variations in folate-pathway enzyme affinity, or differential expression of resistance determinants (36). Because the FICI calculation is directly dependent on the MIC of each component, even moderate inter-strain variability can disproportionately influence the arithmetic mean, whereas the median more accurately reflects the central tendency of the majority of isolates. Importantly, this statistical heterogeneity is consistent with the pharmacokinetic considerations discussed below. If the effective trimethoprim-sulfonamide ratio at the infection site is itself dynamic and variable over time, it is plausible that the *in vitro* interaction pattern may also differ across isolates with distinct susceptibility profiles. Therefore, dispersion in FICI values should not be interpreted solely as methodological variability, but rather as an indication that ratio-dependent synergism is biologically conditional and strain-specific.

Notably, when interaction patterns were evaluated using median FICI values, synergistic interactions (FICI < 1) were detected in substantially more bacteria-combination-ratio settings than when based solely on mean values. This indicates that a limited number of higher FICI observations shifted the arithmetic mean toward indifferent classification in certain cases, despite the majority of isolates exhibiting synergistic behaviour. Therefore, exclusive reliance on mean FICI estimates may underestimate the prevalence of biologically relevant synergism within heterogeneous isolate populations.

Importantly, this variability should not be interpreted solely as methodological noise but rather as a reflection of biological and pharmacodynamic complexity. Such variability therefore likely reflects conditional, strain-specific synergism rather than random experimental variability.

An important observation of this study is that the extent of synergistic interactions identified on the basis of FICI values was largely consistent with the results of the susceptibility testing. Species characterized by lower MIC_50_ and MIC_90_ values and narrower MIC ranges, such as *G. parasuis*, also demonstrated lower median FICI values and tighter interquartile ranges, supporting the biological coherence between baseline susceptibility and interaction strength. In contrast, species with broader MIC distributions displayed greater dispersion in FICI values, indicating more heterogeneous interaction patterns. Based on these findings, a meaningful difference among the investigated potentiated sulfonamide combinations was demonstrated exclusively for *P. multocida* strains, in favour of the TMP-SCP combination.

An additional exploratory analysis of paired isolate-level combination MIC and FICI values further highlighted this complexity. No consistent association was observed between the MIC magnitude of the potentiated sulfonamide combinations and the corresponding interaction outcome. In several cases, isolates with similar combination MIC values displayed markedly different FICI values and categorical interaction patterns. This suggests that, although susceptibility and interaction behaviour are related at a broader biological level, the extent of synergism cannot be reliably inferred from combination MIC values alone. Rather, the interaction profile appears to depend on the relative pharmacodynamic contribution of the two components and on strain-specific biological factors.

From a clinical perspective, it is particularly noteworthy that antagonistic interactions were also detectable at higher sulfonamide ratios. Although these antagonistic classifications were frequently driven by isolated higher FICI values rather than uniform shifts in the central tendency, their occurrence indicates that excessive imbalance between the two components may attenuate sequential pathway inhibition under certain exposure conditions. Although *in vitro* antagonism cannot always be directly extrapolated to clinical outcomes, such interactions may be associated with reduced antimicrobial activity and delayed bacterial clearance, which could be unfavourable from a therapeutic standpoint (40, 41).

These pharmacodynamic observations must be interpreted in light of pharmacokinetic reality. From a pharmacokinetic standpoint, trimethoprim and sulfonamides exhibit distinct disposition characteristics in pigs. Trimethoprim is moderately lipophilic and distributes extensively into tissues, resulting in a relatively large volume of distribution and tissue concentrations that may exceed plasma levels. In contrast, sulfonamides are more hydrophilic and remain largely confined to extracellular compartments. As a consequence, after administration of fixed-ratio formulations, the trimethoprim-sulfonamide concentration ratio in plasma and at infection sites changes over time as a function of differential distribution and elimination kinetics (6, 7, 10).

These pharmacokinetic differences imply that an *in vitro* ratio producing maximal synergism does not necessarily correspond to the predominant *in vivo* exposure ratio at the site of infection. The concentration proportions tested *in vitro* may transiently occur during the dosing interval, but are unlikely to remain constant. Therefore, the present findings should be interpreted as pharmacodynamic characterization rather than as direct evidence of clinical superiority of any specific fixed trimethoprim-sulfonamide ratio in pigs. Integration of PK/PD modelling and dynamic time-kill experiments would be required to determine whether ratio optimisation could translate into improved therapeutic outcomes (42, 43).

From a clinical and diagnostic perspective, the present findings raise the question of whether the exclusive use of a single fixed trimethoprim-sulfonamide ratio in routine *in vitro* susceptibility testing adequately captures the interaction potential of these combinations. Current testing standards are largely based on the historically adopted 1:19 ratio (16). However, our data indicate that synergistic interaction patterns may vary substantially across ratios, and in several bacteria-combination settings synergism was more pronounced at the formulation ratio (1:5) than at the conventional 1:19 proportion. This suggests that reliance on a single testing ratio may underestimate interaction strength for certain pathogens.

At the same time, the detection of antagonistic or indifferent interactions at higher sulfonamide proportions warrants attention. Although *in vitro* antagonism does not automatically translate into clinical treatment failure (38), excessive dominance of one component could theoretically reduce the benefit of sequential pathway inhibition and impair bacteriostatic efficacy under specific exposure conditions. These observations do not justify immediate modification of approved formulations or susceptibility testing standards; however, they support the concept that ratio-dependent evaluation may refine pharmacodynamic characterization and inform future PK/PD-based optimisation strategies. Prospective studies integrating dynamic pharmacodynamic models and *in vivo* exposure data would be required before any modification of clinical recommendations could be considered (42).

The results of the statistical modelling confirmed the validity of the experimental observations. Both the type of drug combination and the applied ratio had a significant effect on FICI values, whereas the absence of a three-way interaction suggests that ratio dependence represents a general phenomenon rather than a feature restricted to specific bacteria-combination pairs. This finding further supports the necessity of evaluating potentiated sulfonamides at different ratios *in vitro*.

Finally, it should be emphasised that the present study was limited to isolates originating from pigs. In other animal species, pathogen ecology, antimicrobial exposure and pharmacokinetic characteristics may differ, potentially altering the nature of combination effects. Accordingly, direct extrapolation of these results to other species should be approached with caution, and similar studies in other food-producing or companion animal species may be warranted.

## 5 Conclusion

In conclusion, the present study provides the first systematic evaluation of ratio-dependent trimethoprim-sulfonamide synergy across multiple bacterial pathogens associated with porcine respiratory disease complex (PRDC). Our results demonstrate that the synergistic activity of these combinations is strongly ratio-dependent and pathogen-specific, and cannot be adequately characterized by a single fixed testing proportion. Among the evaluated ratios, the 1:5 trimethoprim-sulfonamide ratio most consistently produced the strongest synergistic interactions *in vitro*, despite the widespread use of the 1:19 ratio in susceptibility testing.

By analysing three different potentiated sulfonamide combinations against four clinically relevant PRDC-associated species, this study highlights that the magnitude and direction of interaction patterns vary not only between pathogens but also between compound pairs and concentration ratios. These findings confirm that synergism cannot be reliably predicted without considering both the relative MIC values of the individual components and the specific bacterial context. These findings confirm that synergism cannot be reliably inferred from MIC values alone and highlight that interaction outcomes are governed by more complex, ratio-dependent and species-specific pharmacodynamic relationships.

Differences between sulfonamide components were generally limited; however, sulfachloropyridazine showed superior activity as an individual compound against *A. pleuropneumoniae* and *G. parasuis*, and the TMP-SCP combination exhibited a distinct advantage against *P. multocida*. Importantly, synergistic interactions identified by FICI analysis were largely consistent with susceptibility testing results, supporting the biological relevance of the observed interactions. Furthermore, evaluation based on median FICI values revealed synergistic interactions in a greater number of bacteria-combination-ratio settings than mean-based interpretation, indicating that reliance solely on arithmetic means may underestimate the prevalence of synergistic interaction patterns within heterogeneous isolate populations. The detection of antagonistic effects at higher sulfonamide ratios further highlights that interaction patterns may shift unfavourably under markedly imbalanced concentration conditions.

Taken together, these data support the concept that ratio-dependent evaluation may refine the interpretation of *in vitro* susceptibility testing of potentiated sulfonamides and improve pharmacodynamic characterization of combination effects. However, given the dynamic pharmacokinetic behaviour of trimethoprim and sulfonamides in pigs, the in vitro optimal ratio identified in this study should not be interpreted as direct evidence for clinical optimisation without integrated PK/PD confirmation.

Overall, the present work demonstrates that ratio dependence represents a consistent phenomenon across multiple PRDC pathogens and provides a framework for future PK/PD-integrated investigations aimed at optimising combination therapy. Further studies incorporating dynamic pharmacodynamic models and *in vivo* exposure data are warranted to determine the clinical relevance of ratio optimisation and to explore its applicability in other animal species.

## Data Availability

The raw data supporting the conclusions of this article will be made available by the authors, without undue reservation.
