# Supplementary material for: Comparative evaluation of trimethoprim-sulfonamide ratios and synergistic interactions against porcine respiratory pathogens
Source: Front Vet Sci. 2026 Apr 10;13:1805735. doi: 10.3389/fvets.2026.1805735 (PMC13107639; doi:10.3389/fvets.2026.1805735)
Supplement: Supplementary file 1 [file Data_Sheet_1.PDF]

# *Supplementary Material*

**Supplementary Table S1.** MIC values (μg/mL) for the active ingredients trimethoprim (TMP), sulfamethoxazole (SMX), sulfachloropyridazine (SCP), and sulfadiazine (SD), against isolates of *Pasteurella multocida* (*P. multocida*), *Streptococcus suis* (*S. suis*), *Actinobacillus pleuropneumoniae* (*A. pleuropneumoniae*), and *Glaesserella parasuis* (*G. parasuis*).

| Strain number | Bacteria | TMP | SMX | SCP | SD |
|---|---|---|---|---|---|
| 1 | *P. multocida* | 256 | 5120 | 160 | >5120 |
| 2 | | 256 | 5120 | 1280 | >5120 |
| 3 | | 128 | 5120 | 640 | 5120 |
| 4 | | 256 | 5120 | 640 | 5120 |
| 5 | | 256 | 5120 | 1280 | 5120 |
| 6 | | 256 | 5120 | 640 | 5120 |
| 7 | | 256 | 5120 | 640 | 5120 |
| 8 | | 256 | 5120 | 640 | 5120 |
| 9 | | 256 | 5120 | 80 | 10240 |
| 10 | | 256 | 5120 | 80 | 10240 |
| 11 | | 256 | 5120 | 160 | 5120 |
| 12 | | 256 | 5120 | 320 | 2560 |
| 13 | *S. suis* | 64 | 2560 | 2560 | 5120 |
| 14 | | 256 | 640 | 1280 | 160 |
| 15 | | 256 | 160 | 80 | 1280 |
| 16 | | 256 | 640 | 320 | 640 |
| 17 | | 256 | 160 | 80 | 320 |
| 18 | | 256 | 640 | 320 | 2560 |
| 19 | | 256 | 160 | 5 | 160 |
| 20 | | 256 | 160 | 320 | 2560 |
| 21 | | 256 | 640 | 320 | 2560 |
| 22 | | 256 | 640 | 160 | 2560 |
| 23 | | 256 | 640 | 160 | 640 |
| 24 | | 64 | 160 | 80 | 160 |
| 25 | | 64 | 320 | 160 | 10240 |
| 26 | | 256 | 640 | 160 | 10240 |
| 27 | *A. pleuropneumoniae* | 8 | 80 | 40 | 160 |
| 28 | | 8 | 80 | 40 | 80 |
| 29 | | 16 | 5120 | 80 | 2560 |
| 30 | | 0.5 | 5120 | 80 | 5120 |
| 31 | | 4 | 80 | 20 | 160 |
| 32 | | 0.5 | 5120 | 80 | 2560 |
| 33 | | 8 | 160 | 40 | 2560 |
| 34 | | 128 | 80 | 160 | 5120 |
| 35 | | 0.5 | 5120 | 80 | 5120 |

| Strain number | Bacteria | TMP | SMX | SCP | SD |
|---|---|---|---|---|---|
| 36 | | 8 | 80 | 40 | 160 |
| 37 | | 8 | 80 | 20 | 640 |
| 38 | | 2 | 5120 | 40 | 2560 |
| 39 | | 8 | 40 | 80 | 320 |
| 40 | *G. parasuis* | 64 | 2560 | 40 | 1280 |
| 41 | | 128 | 640 | 320 | 1280 |
| 42 | | 2 | 1280 | 160 | 2560 |
| 43 | | 64 | 5120 | 320 | 2560 |
| 44 | | 128 | 2180 | 160 | 2560 |
| 45 | | 128 | 20 | 80 | 160 |
| 46 | | 64 | 10 | 10 | 10 |
| 47 | | 62 | 1280 | 40 | 80 |
| 48 | | 16 | 5120 | 80 | 5120 |
| 49 | | 128 | 1280 | 160 | 2560 |

**Supplementary Table S2.** MIC values for trimethoprim-sulfamethoxazole (TMP-SMX) at drug ratios of 1:5, 1:10, 1:19, and 1:40 (µg/mL), against isolates of *Pasteurella multocida* (*P. multocida*), *Streptococcus suis* (*S. suis*), *Actinobacillus pleuropneumoniae* (*A. pleuropneumoniae*), and *Glaesserella parasuis* (*G. parasuis*).

| Strain number | Bacteria | TMP-SMX | | | |
|---|---|---|---|---|---|
| | | 1:5 | 1:10 | 1:19 | 1:40 |
| 1 | *P. multocida* | 256 | 256 | 128 | 128 |
| 2 | | 256 | 256 | 256 | 128 |
| 3 | | 128 | 256 | 64 | 128 |
| 4 | | 128 | 256 | 128 | 128 |
| 5 | | 256 | 256 | 256 | 128 |
| 6 | | 256 | 256 | 256 | 256 |
| 7 | | 256 | 256 | 256 | 256 |
| 8 | | 256 | 256 | 256 | 256 |
| 9 | | 256 | 256 | 256 | 128 |
| 10 | | 256 | 256 | 128 | 128 |
| 11 | | 256 | 256 | 256 | 128 |
| 12 | | 256 | 256 | 256 | 128 |
| 13 | *S. suis* | 8 | 4 | 4 | 4 |
| 14 | | 32 | 32 | 8 | 8 |
| 15 | | 32 | 16 | 4 | 4 |
| 16 | | 64 | 32 | 32 | 32 |
| 17 | | 128 | 64 | 32 | 8 |
| 18 | | 8 | 16 | 16 | 16 |
| 19 | | 32 | 32 | 16 | 8 |
| 20 | | 64 | 64 | 64 | 64 |
| 21 | | 256 | 256 | 256 | 256 |
| 22 | | 16 | 32 | 16 | 16 |

| Strain number | Bacteria | TMP-SMX | | | |
|---|---|---|---|---|---|
| | | 1:5 | 1:10 | 1:19 | 1:40 |
| 23 | | 32 | 32 | 16 | 16 |
| 24 | | 2 | 2 | 2 | 2 |
| 25 | | 8 | 4 | 2 | 2 |
| 26 | | 16 | 16 | 16 | 16 |
| 27 | *A. pleuropneumoniae* | 2 | 2 | 1 | 2 |
| 28 | | 2 | 4 | 2 | 2 |
| 29 | | 1 | 1 | 1 | 1 |
| 30 | | 1 | 0.5 | 0.5 | 0.5 |
| 31 | | 0.5 | 0.5 | 0.5 | 0.25 |
| 32 | | 2 | 2 | 2 | 2 |
| 33 | | 1 | 1 | 1 | 1 |
| 34 | | 4 | 4 | 2 | 2 |
| 35 | | 2 | 2 | 2 | 2 |
| 36 | | 2 | 1 | 1 | 1 |
| 37 | | 1 | 1 | 0.5 | 0.5 |
| 38 | | 4 | 4 | 4 | 2 |
| 39 | | 2 | 2 | 2 | 0.25 |
| 40 | *G. parasuis* | 8 | 8 | 8 | 2 |
| 41 | | 64 | 32 | 16 | 16 |
| 42 | | 2 | 2 | 2 | 2 |
| 43 | | 32 | 32 | 32 | 32 |
| 44 | | 2 | 2 | 1 | 1 |
| 45 | | 2 | 2 | 1 | 1 |
| 46 | | 2 | 2 | 2 | 2 |
| 47 | | 2 | 2 | 2 | 2 |
| 48 | | 1 | 1 | 1 | 0.5 |
| 49 | | 4 | 2 | 2 | 2 |

**Supplementary Table S3.** MIC values for trimethoprim-sulfachloropyridazine (TMP-SCP) at drug ratios of 1:5, 1:10, 1:19, and 1:40 (μg/mL), against isolates of *Pasteurella multocida* (*P. multocida*), *Streptococcus suis* (*S. suis*), *Actinobacillus pleuropneumoniae* (*A. pleuropneumoniae*), and *Glaesserella parasuis* (*G. parasuis*).

| Strain number | Bacteria | TMP-SCP | | | |
|---|---|---|---|---|---|
| | | 1:5 | 1:10 | 1:19 | 1:40 |
| 1 | *P. multocida* | 128 | 64 | 16 | 4 |
| 2 | | 64 | 64 | 16 | 4 |
| 3 | | 64 | 32 | 16 | 16 |
| 4 | | 32 | 32 | 16 | 4 |
| 5 | | 32 | 32 | 32 | 32 |
| 6 | | 32 | 32 | 32 | 32 |
| 7 | | 64 | 64 | 32 | 16 |
| 8 | | 32 | 32 | 16 | 16 |
| 9 | | 64 | 64 | 16 | 16 |
| 10 | | 32 | 32 | 16 | 16 |

| Strain number | Bacteria | TMP-SCP | | | |
|---|---|---|---|---|---|
| | | 1:5 | 1:10 | 1:19 | 1:40 |
| 11 | | 64 | 64 | 32 | 32 |
| 12 | | 64 | 128 | 128 | 128 |
| 13 | *S. suis* | 4 | 2 | 4 | 2 |
| 14 | | 16 | 16 | 4 | 2 |
| 15 | | 8 | 2 | 2 | 2 |
| 16 | | 32 | 16 | 32 | 16 |
| 17 | | 256 | 64 | 64 | 8 |
| 18 | | 16 | 16 | 16 | 8 |
| 19 | | 32 | 32 | 16 | 8 |
| 20 | | 64 | 64 | 64 | 64 |
| 21 | | 256 | 256 | 256 | 128 |
| 22 | | 32 | 32 | 32 | 16 |
| 23 | | 32 | 32 | 32 | 16 |
| 24 | | 4 | 2 | 2 | 2 |
| 25 | | 4 | 2 | 2 | 2 |
| 26 | | 32 | 16 | 16 | 8 |
| 27 | *A. pleuropneumoniae* | 2 | 4 | 2 | 1 |
| 28 | | 2 | 2 | 2 | 2 |
| 29 | | 4 | 2 | 2 | 0.5 |
| 30 | | 0.5 | 0.5 | 0.5 | 0.5 |
| 31 | | 0.5 | 0.5 | 0.25 | 0.25 |
| 32 | | 2 | 2 | 1 | 0.5 |
| 33 | | 2 | 2 | 2 | 2 |
| 34 | | 8 | 8 | 8 | 2 |
| 35 | | 2 | 2 | 1 | 0.5 |
| 36 | | 2 | 2 | 2 | 0.5 |
| 37 | | 2 | 2 | 2 | 0.5 |
| 38 | | 4 | 2 | 2 | 0.5 |
| 39 | | 2 | 2 | 2 | 0.5 |
| 40 | *G. parasuis* | 8 | 8 | 4 | 0.5 |
| 41 | | 32 | 64 | 32 | 8 |
| 42 | | 2 | 2 | 2 | 2 |
| 43 | | 32 | 16 | 16 | 8 |
| 44 | | 2 | 2 | 2 | 2 |
| 45 | | 1 | 1 | 1 | 0.5 |
| 46 | | 4 | 4 | 2 | 2 |
| 47 | | 2 | 4 | 4 | 2 |
| 48 | | 1 | 2 | 1 | 1 |
| 49 | | 4 | 8 | 8 | 2 |

**Supplementary Table S4.** MIC values for trimethoprim-sulfadiazine (TMP-SD) at drug ratios of 1:5, 1:10, 1:19, and 1:40 (μg/mL), against isolates of *Pasteurella multocida* (*P. multocida*), *Streptococcus suis* (*S. suis*), *Actinobacillus pleuropneumoniae* (*A. pleuropneumoniae*), and *Glaesserella parasuis* (*G. parasuis*).

| **Strain number** | **Bacteria** | **TMP-SD** | | | |
|---|---|---|---|---|---|
| | | **1:5** | **1:10** | **1:19** | **1:40** |
| 1 | *P. multocida* | 256 | 256 | 256 | 256 |
| 2 | | 256 | 256 | 256 | 256 |
| 3 | | 256 | 256 | 256 | 128 |
| 4 | | 256 | 256 | 256 | 256 |
| 5 | | 256 | 256 | 256 | 256 |
| 6 | | 256 | 256 | 256 | 256 |
| 7 | | 256 | 256 | 256 | 256 |
| 8 | | 256 | 256 | 256 | 256 |
| 9 | | 256 | 256 | 256 | 256 |
| 10 | | 256 | 256 | 256 | 256 |
| 11 | | 256 | 256 | 256 | 256 |
| 12 | | 256 | 256 | 256 | 256 |
| 13 | *S. suis* | 4 | 4 | 4 | 4 |
| 14 | | 32 | 32 | 32 | 32 |
| 15 | | 8 | 8 | 4 | 4 |
| 16 | | 16 | 16 | 16 | 16 |
| 17 | | 256 | 128 | 32 | 16 |
| 18 | | 16 | 16 | 8 | 8 |
| 19 | | 64 | 32 | 16 | 16 |
| 20 | | 64 | 64 | 64 | 64 |
| 21 | | 256 | 256 | 256 | 256 |
| 22 | | 16 | 16 | 16 | 32 |
| 23 | | 16 | 16 | 16 | 32 |
| 24 | | 4 | 4 | 2 | 2 |
| 25 | | 4 | 4 | 4 | 4 |
| 26 | | 32 | 16 | 16 | 16 |
| 27 | *A. pleuropneumoniae* | 2 | 2 | 2 | 2 |
| 28 | | 2 | 2 | 2 | 2 |
| 29 | | 2 | 1 | 0.5 | 1 |
| 30 | | 0.5 | 1 | 0.5 | 1 |
| 31 | | 1 | 1 | 0.5 | 0.5 |
| 32 | | 2 | 2 | 2 | 2 |
| 33 | | 2 | 1 | 1 | 1 |
| 34 | | 8 | 4 | 8 | 2 |
| 35 | | 2 | 4 | 2 | 1 |
| 36 | | 2 | 2 | 2 | 1 |
| 37 | | 4 | 2 | 2 | 1 |
| 38 | | 4 | 2 | 4 | 4 |
| 39 | | 2 | 2 | 2 | 2 |
| 40 | *G. parasuis* | 16 | 8 | 8 | 8 |
| 41 | | 64 | 64 | 32 | 32 |

| Strain number | Bacteria | TMP-SD | | | |
|---|---|---|---|---|---|
| | | 1:5 | 1:10 | 1:19 | 1:40 |
| 42 | | 2 | 2 | 2 | 1 |
| 43 | | 32 | 64 | 32 | 32 |
| 44 | | 2 | 2 | 2 | 1 |
| 45 | | 2 | 2 | 1 | 1 |
| 46 | | 4 | 4 | 4 | 4 |
| 47 | | 4 | 4 | 2 | 2 |
| 48 | | 2 | 2 | 1 | 1 |
| 49 | | 8 | 8 | 4 | 4 |

**Supplementary Table S5.** FICI values for combinations of the active ingredient trimethoprim-sulfamethoxazole (TMP-SMX) in ratios of 1:5, 1:10, 1:19, and 1:40 against *Pasteurella multocida* (*P. multocida*), *Streptococcus suis* (*S. suis*), *Actinobacillus pleuropneumoniae* (*A. pleuropneumoniae*), and *Glaesserella parasuis* (*G. parasuis*) isolates. Green indicates synergistic (FICI ≤ 0.5), yellow indicates partial synergistic (0.5 < FICI < 1), orange indicates additive (FICI = 1), blue indicates indifferent (1 < FICI < 4), and red indicates antagonistic (FICI ≥ 4) interactions.

| Strain number | Bacteria | TMP-SMX | | | |
|---|---|---|---|---|---|
| | | 1:5 | 1:10 | 1:19 | 1:40 |
| 1 | *P. multocida* | 1.25 | 1.50 | 0.98 | 1.50 |
| 2 | | 1.25 | 1.50 | 1.95 | 1.50 |
| 3 | | 1.13 | 2.50 | 0.74 | 2.00 |
| 4 | | 0.63 | 1.50 | 0.98 | 1.50 |
| 5 | | 1.25 | 1.50 | 1.95 | 1.50 |
| 6 | | 1.25 | 1.50 | 1.95 | 3.00 |
| 7 | | 1.25 | 1.50 | 1.95 | 3.00 |
| 8 | | 1.25 | 1.50 | 1.95 | 3.00 |
| 9 | | 1.25 | 1.50 | 1.95 | 1.50 |
| 10 | | 1.25 | 1.50 | 0.98 | 1.50 |
| 11 | | 1.25 | 1.50 | 1.95 | 1.50 |
| 12 | | 1.25 | 1.50 | 1.95 | 1.50 |
| 13 | *S. suis* | 0.14 | 0.08 | 0.09 | 0.13 |
| 14 | | 0.38 | 0.63 | 0.27 | 0.53 |
| 15 | | 1.13 | 1.06 | 0.49 | 1.02 |
| 16 | | 0.75 | 0.63 | 1.08 | 2.13 |
| 17 | | 4.50 | 4.25 | 3.93 | 2.03 |
| 18 | | 0.09 | 0.31 | 0.54 | 1.06 |
| 19 | | 1.13 | 2.13 | 1.96 | 2.03 |
| 20 | | 2.25 | 4.25 | 7.85 | 16.25 |
| 21 | | 3.00 | 5.00 | 8.60 | 17.00 |
| 22 | | 0.19 | 0.63 | 0.54 | 1.06 |
| 23 | | 0.38 | 0.63 | 0.54 | 1.06 |
| 24 | | 0.09 | 0.16 | 0.27 | 0.53 |
| 25 | | 0.25 | 0.19 | 0.15 | 0.28 |

| Strain number | Bacteria | TMP-SMX | | | |
|---|---|---|---|---|---|
| | | 1:5 | 1:10 | 1:19 | 1:40 |
| 26 | | 0.19 | 0.31 | 0.54 | 1.06 |
| 27 | *A. pleuropneumoniae* | 0.38 | 0.50 | 0.36 | 1.25 |
| 28 | | 0.38 | 1.00 | 0.73 | 1.25 |
| 29 | | 0.06 | 0.06 | 0.07 | 0.07 |
| 30 | | 2.00 | 1.00 | 1.00 | 1.00 |
| 31 | | 0.16 | 0.19 | 0.24 | 0.19 |
| 32 | | 4.00 | 4.00 | 4.01 | 4.02 |
| 33 | | 0.16 | 0.19 | 0.24 | 0.38 |
| 34 | | 0.28 | 0.53 | 0.49 | 1.02 |
| 35 | | 4.00 | 4.00 | 4.01 | 4.02 |
| 36 | | 0.38 | 0.25 | 0.36 | 0.63 |
| 37 | | 0.19 | 0.25 | 0.18 | 0.31 |
| 38 | | 2.00 | 2.01 | 2.01 | 1.02 |
| 39 | | 0.50 | 0.75 | 1.20 | 0.28 |
| 40 | *G. parasuis* | 0.14 | 0.16 | 0.18 | 0.06 |
| 41 | | 1.00 | 0.75 | 0.60 | 1.13 |
| 42 | | 1.01 | 1.02 | 1.03 | 1.06 |
| 43 | | 0.53 | 0.56 | 0.62 | 0.75 |
| 44 | | 0.02 | 0.03 | 0.02 | 0.04 |
| 45 | | 0.52 | 1.02 | 0.96 | 2.01 |
| 46 | | 1.03 | 2.03 | 3.83 | 8.03 |
| 47 | | 0.04 | 0.05 | 0.06 | 0.09 |
| 48 | | 0.06 | 0.06 | 0.07 | 0.04 |
| 49 | | 0.05 | 0.03 | 0.05 | 0.08 |

**Supplementary Table S6.** FICI values for combinations of the active ingredient trimethoprim-sulfachloropyridazine (TMP-SMX) in ratios of 1:5, 1:10, 1:19, and 1:40 against *Pasteurella multocida* (*P. multocida*), *Streptococcus suis* (*S. suis*), *Actinobacillus pleuropneumoniae* (*A. pleuropneumoniae*), and *Glaesserella parasuis* (*G. parasuis*) isolates. Green indicates synergistic (FICI ≤ 0.5), yellow indicates partial synergistic (0.5 < FICI < 1), orange indicates additive (FICI = 1), blue indicates indifferent (1 < FICI < 4), and red indicates antagonistic (FICI ≥ 4) interactions.

| Strain number | Bacteria | TMP-SCP | | | |
|---|---|---|---|---|---|
| | | 1:5 | 1:10 | 1:19 | 1:40 |
| 1 | *P. multocida* | 4.50 | 4.25 | 1.96 | 1.02 |
| 2 | | 0.50 | 0.75 | 0.30 | 0.14 |
| 3 | | 1.00 | 0.75 | 0.60 | 1.13 |
| 4 | | 0.38 | 0.63 | 0.54 | 0.27 |
| 5 | | 0.25 | 0.38 | 0.60 | 1.13 |
| 6 | | 0.38 | 0.63 | 1.08 | 2.13 |
| 7 | | 0.75 | 1.25 | 1.08 | 1.06 |
| 8 | | 0.38 | 0.63 | 0.54 | 1.06 |
| 9 | | 4.25 | 8.25 | 3.86 | 8.06 |
| 10 | | 2.13 | 4.13 | 3.86 | 8.06 |

| Strain number | Bacteria | TMP-SCP | | | |
|---|---|---|---|---|---|
| | | 1:5 | 1:10 | 1:19 | 1:40 |
| 11 | | 2.25 | 4.25 | 3.93 | 8.13 |
| 12 | | 1.25 | 4.50 | 8.10 | 16.50 |
| 13 | *S. suis* | 0.07 | 0.04 | 0.09 | 0.06 |
| 14 | | 0.13 | 0.19 | 0.08 | 0.07 |
| 15 | | 0.53 | 0.26 | 0.48 | 1.01 |
| 16 | | 0.63 | 0.56 | 2.03 | 2.06 |
| 17 | | 17.00 | 8.25 | 15.45 | 4.03 |
| 18 | | 0.31 | 0.56 | 1.01 | 1.03 |
| 19 | | 32.13 | 64.13 | 60.86 | 64.03 |
| 20 | | 1.25 | 2.25 | 4.05 | 8.25 |
| 21 | | 5.00 | 9.00 | 16.20 | 16.50 |
| 22 | | 1.13 | 2.13 | 3.93 | 4.06 |
| 23 | | 1.13 | 2.13 | 3.93 | 4.06 |
| 24 | | 0.31 | 0.28 | 0.51 | 1.03 |
| 25 | | 0.19 | 0.16 | 0.27 | 0.53 |
| 26 | | 1.13 | 1.06 | 1.96 | 2.03 |
| 27 | *A. pleuropneumoniae* | 0.50 | 1.50 | 1.20 | 1.13 |
| 28 | | 0.50 | 0.75 | 1.20 | 2.25 |
| 29 | | 0.50 | 0.38 | 0.60 | 0.28 |
| 30 | | 1.03 | 1.06 | 1.12 | 1.25 |
| 31 | | 0.25 | 0.38 | 0.30 | 0.56 |
| 32 | | 4.13 | 4.25 | 2.24 | 1.25 |
| 33 | | 0.50 | 0.75 | 1.20 | 2.25 |
| 34 | | 0.31 | 0.56 | 1.01 | 0.52 |
| 35 | | 4.13 | 4.25 | 2.24 | 1.25 |
| 36 | | 0.50 | 0.75 | 1.20 | 0.56 |
| 37 | | 0.75 | 1.25 | 2.15 | 1.06 |
| 38 | | 2.50 | 1.50 | 1.95 | 0.75 |
| 39 | | 0.38 | 0.50 | 0.73 | 0.31 |
| 40 | *G. parasuis* | 1.13 | 2.13 | 1.96 | 0.51 |
| 41 | | 0.75 | 2.50 | 2.15 | 1.06 |
| 42 | | 1.06 | 1.13 | 1.24 | 1.50 |
| 43 | | 1.00 | 0.75 | 1.20 | 1.13 |
| 44 | | 0.08 | 0.14 | 0.25 | 0.52 |
| 45 | | 0.07 | 0.13 | 0.25 | 0.25 |
| 46 | | 2.06 | 4.06 | 3.83 | 8.03 |
| 47 | | 0.28 | 1.06 | 1.96 | 2.03 |
| 48 | | 0.13 | 0.38 | 0.30 | 0.56 |
| 49 | | 0.16 | 0.56 | 1.01 | 0.52 |

**Supplementary Table S7.** FICI values for combinations of the active ingredient trimethoprim-sulfadiazine (TMP-SD) in ratios of 1:5, 1:10, 1:19, and 1:40 against *Pasteurella multocida* (*P. multocida*), *Streptococcus suis* (*S. suis*), *Actinobacillus pleuropneumoniae* (*A. pleuropneumoniae*), and *Glaesserella parasuis* (*G. parasuis*) isolates. Green indicates synergistic (FICI ≤ 0.5), yellow indicates partial synergistic (0.5 < FICI < 1), orange indicates additive (FICI = 1), blue indicates indifferent (1 < FICI < 4), and red indicates antagonistic (FICI ≥ 4) interactions.

| Strain number | Bacteria | TMP-SD | | | |
|---|---|---|---|---|---|
| | | 1:5 | 1:10 | 1:19 | 1:40 |
| 1 | *P. multocida* | 1.13 | 1.25 | 1.48 | 2.00 |
| 2 | | 1.13 | 1.25 | 1.48 | 2.00 |
| 3 | | 2.25 | 2.50 | 2.95 | 2.00 |
| 4 | | 1.25 | 1.50 | 1.95 | 3.00 |
| 5 | | 1.25 | 1.50 | 1.95 | 3.00 |
| 6 | | 1.25 | 1.50 | 1.95 | 3.00 |
| 7 | | 1.25 | 1.50 | 1.95 | 3.00 |
| 8 | | 1.25 | 1.50 | 1.95 | 3.00 |
| 9 | | 1.13 | 1.25 | 1.48 | 2.00 |
| 10 | | 1.13 | 1.25 | 1.48 | 2.00 |
| 11 | | 1.25 | 1.50 | 1.95 | 3.00 |
| 12 | | 1.50 | 2.00 | 2.90 | 5.00 |
| 13 | *S. suis* | 0.07 | 0.07 | 0.08 | 0.09 |
| 14 | | 1.13 | 2.13 | 3.93 | 8.13 |
| 15 | | 0.06 | 0.09 | 0.08 | 0.14 |
| 16 | | 0.19 | 0.31 | 0.54 | 1.06 |
| 17 | | 5.00 | 4.50 | 2.03 | 2.06 |
| 18 | | 0.09 | 0.13 | 0.09 | 0.16 |
| 19 | | 2.25 | 2.13 | 1.96 | 4.06 |
| 20 | | 0.38 | 0.50 | 0.73 | 1.25 |
| 21 | | 1.50 | 2.00 | 2.90 | 5.00 |
| 22 | | 0.09 | 0.13 | 0.18 | 0.63 |
| 23 | | 0.19 | 0.31 | 0.54 | 2.13 |
| 24 | | 0.19 | 0.31 | 0.27 | 0.53 |
| 25 | | 0.06 | 0.07 | 0.07 | 0.08 |
| 26 | | 0.14 | 0.08 | 0.09 | 0.13 |
| 27 | *A. pleuropneumoniae* | 0.31 | 0.38 | 0.49 | 0.75 |
| 28 | | 0.38 | 0.50 | 0.73 | 1.25 |
| 29 | | 0.13 | 0.07 | 0.03 | 0.08 |
| 30 | | 1.00 | 2.00 | 1.00 | 2.01 |
| 31 | | 0.28 | 0.31 | 0.18 | 0.25 |
| 32 | | 4.00 | 4.01 | 4.01 | 4.03 |
| 33 | | 0.25 | 0.13 | 0.13 | 0.14 |
| 34 | | 0.07 | 0.04 | 0.09 | 0.03 |
| 35 | | 4.00 | 8.01 | 4.01 | 2.01 |
| 36 | | 0.31 | 0.38 | 0.49 | 0.38 |
| 37 | | 0.53 | 0.28 | 0.31 | 0.19 |
| 38 | | 2.01 | 1.01 | 2.03 | 2.06 |

| **Strain number** | **Bacteria** | **TMP-SD** | | | |
|---|---|---|---|---|---|
| | | **1:5** | **1:10** | **1:19** | **1:40** |
| 39 | | 0.28 | 0.31 | 0.37 | 0.50 |
| 40 | *G. parasuis* | 0.31 | 0.19 | 0.24 | 0.38 |
| 41 | | 0.75 | 1.00 | 0.73 | 1.25 |
| 42 | | 1.00 | 1.01 | 1.01 | 0.52 |
| 43 | | 0.56 | 1.25 | 0.74 | 1.00 |
| 44 | | 0.02 | 0.02 | 0.03 | 0.02 |
| 45 | | 0.08 | 0.14 | 0.13 | 0.26 |
| 46 | | 2.06 | 4.06 | 7.66 | 16.06 |
| 47 | | 0.31 | 0.56 | 0.51 | 1.03 |
| 48 | | 0.13 | 0.13 | 0.07 | 0.07 |
| 49 | | 0.08 | 0.09 | 0.06 | 0.09 |
